# CpG Oligodeoxynucleotides for Anticancer Monotherapy from Preclinical Stages to Clinical Trials

**DOI:** 10.3390/pharmaceutics14010073

**Published:** 2021-12-28

**Authors:** Zhongkun Zhang, Jimmy Chun-Tien Kuo, Siyu Yao, Chi Zhang, Hira Khan, Robert J. Lee

**Affiliations:** 1 Division of Pharmaceutics and Pharmacology, College of Pharmacy, The Ohio State University, 500 W 12th Avenue, Columbus, OH 43210, USA; zhang.5763@osu.edu (Z.Z.); kuo.249@osu.edu (J.C.-T.K.); zhang.9395@osu.edu (C.Z.); hirakhan464@gmail.com (H.K.); 2 Department of Food Science and Technology, The Ohio State University, 110 Parker Food Science and Technology Building, 2015 Fyffe Road, Columbus, OH 43210, USA; yao.806@osu.edu; 3 Department of Pharmacy, Abbottabad University of Science and Technology, Havelian, Abbottabad 22500, Pakistan

**Keywords:** CpG oligonucleotide, toll-like receptor 9, immunotherapy

## Abstract

CpG oligodeoxynucleotides (CpG ODNs), the artificial versions of unmethylated CpG motifs that were originally discovered in bacterial DNA, are demonstrated not only as potent immunoadjuvants but also as anticancer agents by triggering toll-like receptor 9 (TLR9) activation in immune cells. TLR9 activation triggered by CpG ODN has been shown to activate plasmacytoid dendritic cells (pDCs) and cytotoxic T lymphocytes (CTLs), enhancing T cell-mediated antitumor immunity. However, the extent of antitumor immunity carried by TLR agonists has not been optimized individually or in combinations with cancer vaccines, resulting in a decreased preference for TLR agonists as adjuvants in clinical trials. Although various combination therapies involving CpG ODNs have been applied in clinical trials, none of the CpG ODN-based drugs have been approved by the FDA, owing to the short half-life of CpG ODNs in serum that leads to low activation of natural killer cells (NK cells) and CTLs, along with increases of pro-inflammatory cytokine productions. This review summarized the current innovation on CpG ODNs that are under clinical investigation and explored the future direction for CpG ODN-based nanomedicine as an anticancer monotherapy.

## 1. Antitumor Immunity by CpG ODN-TLR9 Activation

It is known that bacterial DNA is a potent stimulus to immune systems, while the most active sequence in bacterial DNAs is the unmethylated CpG di-deoxynucleotide motif. Toll-like receptor 9 (TLR9), a pattern recognition receptor expressed on endosomal surfaces of immune cells, recognizes not only unmethylated CpG di-deoxynucleotide motifs in bacterial or viral DNAs but also synthetic CpG oligodeoxynucleotides (CpG ODNs). TLR acts through the Myeloid differentiation primary response 88 (MyD88) and the NF-κB pathway and triggers the activation of naïve T cell repertoires in adaptive immune responses [1]. The binding of CpG ODNs to TLR9 induces a conformational change of TLR9, which is thought to result in differential recruitment of one or more TIR domain-containing adaptors. The activated TLR9-TIR complex will further mediate downstream signaling to activate MyD88, TIR domain-containing adaptor-inducing IFN-beta (TRIF), TIR-containing adaptor protein/MyD88-adaptor-like (TIRAP/MAL), or TRIF-related adaptor molecule (TRAM) [2]. Generally, all TLR activations will eventually activate transcription factor NF-κB through either MyD88-dependent or MyD88-independent mechanisms [2]. Unlike TLRs expressed on the cell surfaces, TLR9, an endosomal TLR, could utilize MyD88 to recruit interferon regulatory factor 7 (IRF7) and form the MyD88-IRF7 complex [3,4]. This complex is further phosphorylated by interleukin-1 receptor-associated kinase 1 (IRAK1) and inhibitor of nuclear factor-kappa B kinase (IKKa) and translocated into the nucleus to induce high expression of type I interferons (IFN).

At the cellular level, TLR9 activation triggers the production of pro-inflammatory cytokines through either the MyD88-IRF7 pathway or the MyD88-NFκB pathway. CpG ODNs have been demonstrated to activate plasmacytoid dendritic cells (pDCs) to secrete type I IFNs and express a high level of co-stimulatory molecules for T cell binding and maturation, such as CD80 and CD86 [5]. At the time of pDC activation, a wide range of cytokine secretions, such as IL-2, IL-12, or IFNγ, has been demonstrated to act in concordance to eventually lead to natural killer cells (NK cells) activation and T cell expansion [6].

Despite the controversial roles of cytokines in tumor suppression and immune escape, it is believed that antitumor immunity has mainly relied on IL-12 and IFNγ mediated CD4+ T helper cell activation as well as cytotoxic T lymphocyte (CTL) expansion after pDC presentation [7,8]. Type 1 T helper cells (Th1 cells) are activated by the signature cytokine IFNγ [9]. The antitumor immunity by Th1 cells relies on indirect activation of NK cells and tumor-suppressing CTLs triggered by antigen-presenting cells (APCs). NK cells and CTLs could further release cytotoxic granules containing perforin and granzymes that lead to tumor cell lysis [10,11]. Type 2 T helper cells (Th2 cells) are another polarized T helper cell repertoire that is activated by IL-4, IL-5, and IL-13 production [12]. Th2 cells-mediated antitumor immunity involves the recruitment of eosinophils to the tumor microenvironment (TME) through IL-4 and IL-13 feedback loops, although the anti-inflammatory cytokines produced by Th2 cell polarization may have controversial effects on regulatory T cell (Treg) induction, which may hamper the Th1 antitumor immunity [13,14]. Lastly, type 17 T helper cells (Th17 cells) are polarized through TGFβ, IL-6, and IL-17 [12], and their role in TME remains controversial. The pro-tumor activity mediated by Th17 cells has been confirmed through angiogenesis and the production of anti-inflammatory cytokines [15]. Despite studies demonstrating the pro-tumor activities by Th17 cells, Th17 cells can directly eradicate tumor cells through IFNγ production [16]. In addition, IL-17 secreted by Th17 cells could activate CTLs and convert them into IFNγ-producing effector T cells, in concordance with Th1-mediated antitumor immunity [17,18].

It is also known that autologous antitumor immunity could be achieved by silencing regulatory immune cells such as Tregs and myeloid-derived suppressor cells (MDSCs) [19,20]. It is known that Tregs are involved in tumor progression by silencing the functions of CTLs through multiple mechanisms. After Tregs are chemically attracted to TME by chemokine gradients, Tregs could inhibit APC maturation through inhibitory binding between CTLA-4 and CD80/86 [21]. Moreover, Tregs are demonstrated to highly express high-affinity IL-2 receptors that dominantly consume IL-2, a cytokine essential to effector T cells proliferation [22]. It is also suggested that cytotoxic granules produced by Tregs could directly kill CTLs [23].

Activated MDSCs also participate in tumor progressions through multiple aspects in TME, such as facilitating immune escape, promoting angiogenesis, pre-metastatic niche formation, and epithelial-mesenchymal transition [24,25,26]. Besides, MDSCs are known to mediate immune suppression by targeting T cells. MDSCs could produce high levels of nitric oxide, reactive oxygen species, and peroxynitrites to inhibit T cell proliferation and induce apoptosis [27]. Conversely, MDSC-secreted IL-10 and TGFβ are implicated in effector T cell suppression and Treg induction [28,29,30].

## 2. CpG ODN Classification and Antitumor Efficacy

CpG ODNs are short, single-stranded synthetic nucleic acids that contain unmethylated cytosine-guanine (CG) di-nucleotides in a specific base-pair sequence, called CpG motifs. Since CpG ODNs are rapidly degraded by nucleases after being administered into the systemic circulation, the development of CpG ODN has been focused on delivery systems that are resistant to nucleases [31,32,33,34]. Synthetic CpG ODNs possess partially or completely phosphorothioate backbones, instead of natural phosphodiester backbones, and sometimes a poly-G tail at either or both 3′ and 5′ ends. CpG ODNs have been demonstrated to strongly activate human peripheral blood mononuclear cells (PMBCs) proliferation and pro-inflammatory cytokine secretion [35]. Based on their specific chemical modifications and immunostimulatory effects, CpG ODNs can be classified into three classes: class A (type D), class B (type K), and class C.

Class A (type D) CpG ODNs contain a central palindromic phosphodiester CpG sequence and a PS-modified 3′ poly-G tail. The poly-G tails on CpG-A ODNs could form intermolecular G-quadruplexes or concatemers, forming highly ordered structures. Reports have shown that class A CpG ODNs exhibit enhanced molecular stability and increased endosomal uptake resulting in a high level of IFNα production and pDC maturation, but with no effects on B cell regulations [36,37]. Class A CpG ODNs could also indirectly activate NK cells through cytokine signaling [38]. Research has shown that class A CpG ODNs can retain in the early endosome longer, which makes it rapidly interact with MyD88/IRF-7 through endosomal TLR activation exclusively and effectively trigger IFNα production [39]. Established class A CpG ODNs include ODN 1585, ODN 2216, ODN 2336, and CMP-001 (also known as vidutolimod).

In general, class A CpG ODNs are superior for enhancing memory CTL responses and real-time CTL cytotoxicity to class B CpG ODNs [40]. In a study of ODN 1585, scientists have shown that ODN 1585-mediated TLR activation could successfully induce dendritic cell migration in lymph nodes and IL-12 elevation in peripheral blood in a melanoma xenograft mouse model [41]. They also demonstrated that macrophages rapidly infiltrated into TME but not pDCs [41]. Similar activity was observed in the melanoma syngeneic murine model, where scientists further demonstrated that NK cells contributed to the antitumor immunity led by ODN 1585 treatments [42]. In addition, ODN 1585-mediated CTL activation and IL-12 production were also observed in a study using the MHC-I-deficient HPV16-associated lung tumor model [43]. CTL and NK activations were also observed using a melanoma syngeneic murine model treated with ODN 2216 [44]. Another study verified that class A CpG ODNs enhanced NK cell cytotoxicity against Tu167 and K562 cell lines in vitro [45]. However, a few reports have indicated that CpG ODN treatments may not result in B cell-based antitumor activities [46,47]. Contrarily, TNFα-secreting B cells induced by ODN 2216 may show resistance to certain chemotherapies in mice models [46].

Class B (type K) CpG ODNs contain a complete phosphorothioate backbone with one or more 6-mer CpG motifs. The fully phosphorothioated backbone greatly prevents nuclease digestion, substantially enhancing half-life from less than 10 min to 30–60 min in vivo without delivery platforms [48,49]. Class B CpG ODNs have been demonstrated to trigger pDC differentiation and stimulate robust B cell activations but less NK cell activations compared to class A CpG ODNs [50,51]. Established class B CpG ODNs include ODN 1668, ODN 1826 (ODN 2138), ODN 2006 (ODN 7909/ ODN 2137/ PF-03512676/Agatolimod), ODN 2007, ODN BW006 (ODN 684), ODN DSL01, Litenimod (Li-28/CpG 28), and CpG 685 (GNKG-168).

Class B CpG ODNs were shown to be effective Th1 activators but not NK cell direct-activators. ODN 1668 demonstrated a potent antitumor activity through Th1-mediated CTL activation in a pulmonary metastasis syngeneic mouse model, whereas the NK cell activity was not dependent on ODN 1668 but the cationic liposomal formulation [52]. Another study showed that the immunostimulatory nano-hydrogel encapsulating ODN 1668 could promote Th1 cytokine production [53], suggesting the great potential of immunostimulatory delivery systems encapsulating CpG ODNs in cancer therapeutics. Similar antitumor responses of ODN 1668 and other class B CpG ODNs were observed in murine melanoma, bladder cancer, fibrosarcoma, and lung cancer models [54,55,56,57]. Although CpG 685 was identified as a class B CpG ODN, scientists surprisingly found that CpG 685 exhibited certain characteristics of class C CpG ODNs, facilitating Th1 activation and NK cell proliferation [57]. The class C-biasing biological functions in CpG 685 were demonstrated not only in solid tumor models but also in B cell chronic lymphocytic leukemia models (B-CLL) [58]. Conversely, CpG 685 was shown to have anticancer activities through c-MYC/p53/BAX-mediated apoptosis in ALL and B-CLL [59,60]. A similar apoptotic mechanism was observed in ODN 7909 treatments where the CpG ODN could promote radio-sensitization via p53-dependent apoptosis pathway and enhance Th1 cytokine production in combination with radiotherapy [61,62]. Different from class A CpG ODNs, multiple studies demonstrated that class B CpG ODNs could have the potential of regulating PD-1/PD-L1 expressions [63,64,65].

Class C CpG ODNs combine the features of both class A and class B CpG ODNs. A class C CpG ODN is composed of a fully phosphorothioate-based backbone and palindromic CpG motifs. Consistent with the immunostimulatory effects carried out by class A and class B CpG ODNs, class C CpG ODNs have been demonstrated to strongly trigger B cell activation as well as IFNα production [66]. Established class C CpG ODNs include ODN 2395, ODN M362, ODN DSL03, SD-101, and DV281. Class P CpG ODNs contain two separate palindromic regions that substantially elevated the IFNα-inducing activity compared with class C CpG ODNs, with other cytokine levels remaining similar to other established CpG ODN classes [67].

It has been shown that tumor-bearing mice treated with ODN 2395 had substantial DC accumulations in spleens and tumors, associating with antitumor activities [68]. ODN 2395 has also been demonstrated to potentiate mononuclear cell cytotoxicity in another syngeneic melanoma mouse model [69]. The most recent result from TriSalus indicated that ODN 2395 played an important role in reducing liver MDSC and biasing M1 macrophage polarization in a murine liver metastasis model [70]. A novel class C CpG ODN HP06T07 has been shown to increase Th1 cytokine productions and B cell activation in vitro [71]. HP05T07 was also shown to enhance tumor infiltration of T cells, NK cells in a syngeneic colorectal murine model [71]. Similar trends were also observed in the study of SD-101, which promoted accumulated CTLs, TNFα, and IFNγ independent of CD4 T cell activity [72]. SD-101 was also reported to regulate immune checkpoints and overcome resistances to PD-1 blockade, sharing similar characteristics with class B CpG ODNs [72]. In a study comparing the immunostimulatory activity of all three types of classical CpG ODNs, class C CpG ODNs demonstrated higher efficiency in mediating NK cell-dependent cytotoxicity compared to class A and class B CpG ODNs, although class A CpG ODNs still produced the highest IFNγ in favoring of CTL activation [66]. In recent studies, class C CpG ODNs were superior in B cell stimulation, NF-κB activation, and memory T cell activation to class B CpG ODNs [63].

## 3. Innovative CpG ODN

While classical CpG ODNs exhibit linear sequences with phosphorothioate modification, the development of CpG ODNs has been innovated by modifying sequence shape, base pairs, formulations, or drug conjugations, which eventually enhance the antitumor immunity and prevent CpG ODNs from nuclease degradation.

Lefitolimod (also known as MGN1703) is a small DNA molecule named double-stem loop immunomodulator (dSLIM) with 116 nucleotides that is under clinical development [73]. The molecule exhibits a dumbbell-shaped structure by stretching reverse complementary DNA and forming a 28 base pair double-stranded midsection, flanked at both ends by a single-stranded loop with 30 nucleotides [74]. The nucleotide sequence was maintained by phosphodiester bonds with three CG motifs on each single-stranded loop structure, and no unnatural components were present in the molecule. It is also noticeable that the dumbbell-shaped structure with CpG-rich motifs has been demonstrated to induce high antibody production and B-cell differentiation, whereas the linear phosphorothioated structure with the same sequence of dumbbell-shaped structure exhibited much higher pro-inflammatory factors, especially IL-12 and IFNγ, which potentially promote antitumor immunity carried out by Th1 activation [75,76]. Nonetheless, IFNα induced by MGN1703 has been reported to amplify both B cell and NK cell activations that also result in antibody productions against tumor cell antigens in concordance with NK cell-mediated ADCC [74]. In addition, the dumbbell-shaped structure with CpG motifs on the loops showed the highest IL-12 and IFNα production compared with the single loop structure, the CpG motif on one side of the dumbbell, or the CpG motif on the linear sequence [76]. Although the cytokine production level of MGN1703 is not superior to ODN 2216 or ODN 7909, MGN1703 showed the least liver and spleen toxicity compared with phosphorothioated traditional CpG ODN classes [77,78,79]. Therefore, the take-home message is that the unmodified CpG ODNs with structural innovation such as dumbbell-shaped are preferred for the clinical investigation whereas phosphorothioated sequences should be avoided due to the several toxic effects taken place in clinical failures.

Another innovation in CpG sequence-based TLR agonists included immunomodulatory oligonucleotides (IMOs), which consist of cytosine phosphate-2′-deoxy-7-deazaguanosine dinucleotide (CpR) motifs [80]. The replacement of 7-deazaguanisine in place of guanine in the unmodified CpG motifs could not only enhance TLR9 activation but also spontaneously activate TLR7 and TLR8, which could synergize Th1 immune responses [81]. IMOs are demonstrated as strong inducers of IFNα but much less potent in TNFα and IL-12 induction [81]. IMOs also showed B cell activation in the manner of increasing IFNα and IP-10 secretions [82]. Tilsotolimod (also known as IMO-2125) and IMO-2055 have been developed based on the rationale of CpR motifs. Preclinical studies have shown that tilsotolimod and IMO-2055 not only induced pro-inflammatory cytokines and lymphocyte activation but also had therapeutic potential in various tumor models [83,84]. IMO-2055 showed a significant increase of tumor-specific CTLs in situ of CT26, which could be amplified in combination with ipilimumab treatment [83]. Superior to IMO-2055, intratumoral injection of tilsotolimod led to increased CD3+ T cell infiltration and CTL response both in situ and remote tumors, and removal of CD4+ T cells showed more robust antitumor responses by IMOs due to the depletion of Tregs [85]. Treatment of IMOs also showed immune checkpoint upregulations (IDO-1, PD-L1, CEACAM1), suggesting that the complete antitumor responses of IMOs may require the combination with immune checkpoint inhibitors [85].

CMP-001 is another CpG-based TLR9 agonist under clinical studies in patients with NSCLC and melanoma. CMP-001 is a virus-like particle encapsulating class A CpG ODN with Qβ bacteriophage capsid proteins [65]. Preclinical studies indicated that CMP-001 had remarkable antitumor immunity not only in combination with immune checkpoint inhibitors but also as a monotherapy in multiple tumor models [65,86]. However, T cell-dependent antitumor immunity activated by CMP-001 was highly dependent on the presence of anti-Qβ antibodies that would facilitate pDCs to rapidly recognize and uptake CMP-001 based on the structure [87].

Delivery of oligonucleotides or other nucleic acids using artificial non-viral nanoparticles is a promising strategy in anticancer therapeutics [88]. Spherical nucleic acid (SNA) is a nanoparticle-based delivery strategy that consists of a lipophilic core nanoparticle coated in densely packed nucleic acids that are linked by metal-thiol interaction. Cavrotolimod (also known as AST-008) is an SNA consisting of TLR9 agonist densely packed on the surface of a liposomal nanoparticle core. Cavrotolimod exhibits properties that distinguish itself from those TLR9 agonists with the linear structure under the same sequence, which includes increased cellular uptake, improved pharmacokinetics, and biodistribution [89]. Cavrotolimod has also been demonstrated to induce potent Th1 immune responses and antitumor activities as a monotherapy or in combination with immune checkpoint inhibitors in various models [90].

Toll-like receptor agonist-antibody conjugate (TRAAC) is another delivery platform for the systemic administration of TLR agonists. In this platform, scientists have confirmed that B cells played important roles in responses to checkpoint inhibitors in many tumor models [91]. TAC-001 is a TRAAC composed of a linear monomeric CpG ODNs with non-aggregation property, which resembles class B CpG ODNs, conjugated to an anti-CD22 antibody that specifically targets CD22-expressed B cells [92]. Treatment with TAC-001 showed an increased level of CD86 and early immune activation compared with treatment with its CpG ODN motif alone. B cell-specific activation was also observed in TAC-001 treated human PBMCs with upregulated expressions of CD19 and CD40, but CD80+ pDC and CD85+ monocyte activations were not observed in TAC-001 treatments, compared with single treatments using anti-CD22 antibody or CpG ODN. Treatments with TAC-001 also showed a decrease in IL-10-secreting regulatory B cells (Bregs) with increased effector T cell functions (IFNγ + CD4+ T cells and Granzyme + CTLs).

## 4. CpG ODN Monotherapy in Anticancer Clinical Investigation

Abundant clinical trials demonstrated the maintenance therapeutic efficacy of CpG ODNs along with radiation, chemotherapy, or immune checkpoint inhibitors. The rationale of these combination therapies is based on the enhanced TLR9 stimulation and tumor-specific CTLs activity carried out by cytotoxic treatments as well as immune checkpoint blockades in TME. However, the solid antitumor immunity from CpG ODNs monotherapy has been hidden from the rapid clinical development of CpG ODN combination therapies with other anticancer agents. Anticancer applications of CpG ODN as monotherapy in clinical trials have been summarized in Table 1.

### 4.1. CpG 7909

CpG 7909 is the earliest CpG ODN to enter clinical studies. The first phase I/II study was performed to determine the maximum tolerated dose (MTD), safety profile, antitumor activity, pharmacokinetics, and immunologic effects in patients with stage IV renal cell carcinoma (NCT00043407). Thirty-nine patients were enrolled and received CpG 7909 subcutaneously at doses of up to 0.81 mg/kg. Two patients achieved partial responses for over 30 months. CpG 7909 was tolerable as a single-agent treatment and exhibited modest antitumor activity [93]. In another phase I dose-escalating trial in patients with cutaneous T-cell lymphoma (NCT00043420), 28 patients received up to 0.36 mg/kg of CpG 7909 subcutaneously. The best overall response rate was 32%, with three complete responses and six partial responses, as determined by both Composite Assessment of Index Lesion Severity and Physician Global Assessment [94]. Patients (*n* = 23) who were previously enrolled in phase I trial for the dose-escalating study further received CpG 7909 intravenously at 0.01–0.64 mg/kg (NCT00043368). NK cell activities were observed in patients along with increased antibody-dependent cellular cytotoxicity activity. The mean ratio of NK cell concentrations post-treatment to pre-treatment was 1.44 (95% CI = 0.94–1.94) on day 2 and 1.53 (95% CI = 1.14–1.91) on day 42. However, no clinical response was observed at day 42 while two patients developed later responses [95]. Similar immunostimulatory effects of CpG 7909 were observed in a phase II trial of 24 patients with metastatic melanoma (NCT00070642). Patients receiving CpG 7909 subcutaneously at doses of 10 or 40 mg weekly exhibited increased frequencies of both melanoma-associated-specific CTLs and NK cells in the sentinel lymph node, which correlated with CpG 7909-induced pDC maturation [96]. The application of CpG 7909 in blood cancer was examined in a phase I study in 41 patients with chronic lymphocytic leukemia (NCT00233506). No clinical response was reported when CpG 7909 was administered intravenously (1.05 mg/kg) or subcutaneously (0.45 mg/kg) [102]. However, intravenously administered CpG 7909 had transient but significant CD20 expressions along with relatively high NK cell and T cell activations compared with that through subcutaneous administration [102]. These results provided insights that intravenous administration of CpG 7909 or other TLR9 agonists against blood cancers would be preferred to sensitize leukemic cells for antibody therapies.

### 4.2. Litenimod

In a phase I trial of patients with recurrent glioblastoma (GBM), litenimod was well tolerated up to 20 mg per injection by convection-enhanced delivery. Minor responses were observed in two patients, and the overall median survival was 7.2 months as reported. The most common toxicities were lymphopenia, mild fever, seizures, and transient neurological worsening [97]. In a further phase II trial in patients with recurrent glioblastoma (GBM), 31 patients received local treatments of 20 mg litenimod (NCT00190424). The reported progression-free survival was 19% at 6 months with one partial response and three minor responses observed. The median overall survival was 28 weeks. Litenimod has been demonstrated to have modest activity on the 6-month PFS in patients, but its efficacy did not meet the targeted PFS benefit [98].

### 4.3. IMO-2055

The safety and efficacy of IMO-2055 as a single agent was evaluated in a phase II trial in patients with recurrent or metastatic renal clear cell carcinoma (NCT00729053). Ninety-two patients were enrolled and treated with IMO-2055 subcutaneously at 0.16 or 0.64 mg/kg. The best overall objective response rate of 0.64 mg/kg IMO-2055 treatment was 4.8% in patients with previous treatments and 4.3% in patients with treatment-naïve. No overall objective response was observed in patients treated with 0.16 mg/kg IMO-2055. Overall survival in patients at 1 year was over 60% regardless of the dose regimen or previous treatments. Median time to disease progression was over 100 days in patients with previous treatments (0.16 and 0.64 mg/kg) and treatment-naïve (0.64 mg/kg), and 59 days for treatment-naïve patients treated with 0.64 mg/kg IMO-2055. Serious adverse events were lower than 50% regardless of the dose regimen and previous treatments.

### 4.4. GNKG168

The safety, tolerability, efficacy, and pharmacokinetics of GNKG168 were evaluated in two phase I studies in patients with relapsed or refractory B-CLL (NCT01035216) and patients with r/r ALL and AML (NCT01743807). Patients were administered GNKG168 intravenously with a maximum dose of 0.25 mg/kg. However, the trials have been suspended due to the support termination from the sponsor.

### 4.5. MGN1703

A phase II randomized, double-blind, multi-center study was performed to evaluate the efficacy and safety of MGN1703 as maintenance therapy for patients with advanced colorectal carcinoma with disease control after first-line standard chemotherapies (NCT01208194). The median OS was 22.6 months for patients treated with MGN1703 and 15.1 months for patients treated with placebo, with an HR of 0.63 in the whole study population. A subgroup of patients randomized into the study with confirmed RECIST response showed a median overall survival of 24.5 months with an HR of 0.40. Later, the study showed that HR for patients with normalized CEA or with activated NKT cells were 0.69 and 0.43, suggesting that patients with activated NK cells would receive greater benefits from MGN1703 treatments [99]. Based on the previous results, patients with mCRC and objective response after standard induction therapies were further randomized into a phase III study to investigate the maintenance effect of MGN1703 in comparison with usual maintenance treatments (NCT02077868). Unfortunately, the overall survival was not improved with MGN1703 compared with usual maintenance therapies in patients with metastatic colorectal cancer in the phase III IMPALA trial.

Another phase II trial was performed to evaluate the efficacy and safety of MGN1703 as the maintenance therapy in patients with small-cell lung cancer who have achieved a partial response to platinum-based first-line therapy (NCT02200081). MGN1703 exhibited a favorable safety profile and pharmacodynamic assessment confirmed the mode-of-action showing a clear activation of monocytes and production of IFNγ-induced protein 10 (IP-10). The median OS was 22.0 months in patients treated with MGN1703, compared with 21.9 months in patients treated with standard of care (HR, 1.12; 95% CI, 0.91–1.38; *p* = 0.2765), which failed to meet the primary endpoint of the trial. However, OS signals were observed in two subgroups of patients with chronic obstructive pulmonary disease (COPD) and with a low number of activated B cells. Patients with COPD showed HR of 0.48 (95% CI: 0.20–1.17), and patients with the low number of activated B cells showed HR of 0.53 (95% CI: 0.26–1.08). The finding suggested that treatments of MGN1703 or other TLR9 agonists may favor the tumor environment with a lower CD86+ B cell population, especially regulatory B cells [100].

### 4.6. IMO-2125

A phase Ib dose-escalating study was performed in patients with advanced melanoma and other refractory solid tumors (NCT03052205). A total of 54 patients were enrolled and treated with IMO-2125 intratumorally at doses up to 32 mg. No dose-limiting toxicity was observed during the treatment course. The most common treatment-related adverse events were pyrexia, fatigue, chills, nausea, and vomiting. Biopsies of injected tumors at 24 h showed increased activation of Type I IFN pathway, IFNγ expression, expression of multiple immune checkpoints, and upregulation of MHC class I/II, (i.e., PD-1, LAG3). Thirty-four percent of evaluable patients have achieved the best overall response with stable disease (SD). Specifically, three patients achieved SD in the melanoma cohort (N = 16) with one additional patient who had a 35% tumor reduction with no confirmatory scan [101]. IMO-2125 was generally well tolerated and induced Th1-type immune activation in the tumor microenvironment. However, the overexpression of immune checkpoints induced by IMO-2125 in the tumor environment may block the antitumor activity of tumor-infiltrating CTLs and NK cells during long-term treatments. A randomized phase II trial in patients with pT3-4 cN0M0 melanoma (INTRIM) dosed with IMO-2125 intradermally is in progress (NCT04126876).

### 4.7. AST-008

The early data from a phase I study suggested that AST-008 was safe and well-tolerated with no dose-limiting adverse events noted. Th1-biased immune responses with pro-inflammatory cytokines were consistently induced in health volunteers across the treatment course. Specifically, healthy volunteers treated with AST-008 exhibited a 9.5-fold increase in activated T cells and a 3.5-fold increase in activated NK cells [89]. Dose-dependent increases in NK cells and CD8+ T cells peripherally were also observed in a phase Ib/II trial where patients with advanced solid tumors were treated with AST-008 intratumorally as a single agent or in combination with pembrolizumab (NCT03684785).

## 5. Discussion and Conclusions

Despite the wealth of preclinical research in CpG ODN antitumor immunity, none of the CpG ODN-derivatives have been approved as front-line anticancer treatments [103]. Current CpG ODN monotherapies hardly trigger significant cellular immune responses, leading to low antitumor activities [104]. Therefore, the clinical applications for CpG ODN against cancer are still limited to the local administration in combination with chemotherapies or antibodies [105,106,107]. However, chemotherapies are often associated with high cytotoxicity that causes dose-dependent damage to normal cells even if been administered locally to reduce the systemic side effects. Conversely, the anticancer activities of antibody drugs by either positively or negatively regulating T cell activations are limited to “hot tumors” with abundant tumor-infiltrating immune cells, whereas “cold tumors” with less immune cell infiltration in most senior patients showed minor response to such therapeutic strategies [108].

CpG ODN monotherapy can be a promising antitumor strategy with low side effects by enhancing the delivery method to achieve systemic immune response and by optimizing ODN sequence to bypass the negative regulatory circuits in NK cells and T cells. Considering CpG ODNs being easily degraded by nucleases before being recognized by APCs, delivery platforms such as lipid nanoparticles should be developed to increase drug retention during treatments, therefore achieving consistent immune activation and antitumor immunity. An efficient delivery platform would also allow CpG ODNs to be used in systemic administration instead of intratumoral administration, which improves the therapeutic efficacy in patients with metastatic cancer stages. From preclinical and clinical results of CpG ODN therapies, an unparalleled increase of pro-inflammatory cytokine production was observed due to synergistic activations of NK cells, T cells, and B cells. Although the antitumor immunity carried out by CpG ODN therapy is mainly attributed to the NK cell and T cell cytotoxicity, it is noticeable that the B cell population plays an important role in regulating CpG ODN-induced antitumor activity. The activations of B cells, especially regulatory B cells, are highly correlated with poor antitumor response in both preclinical and clinical studies [100,109]. The partial immune activation of NK cells and T cells without B cells can be achieved by designing CpG ODNs with structures biasing the characteristics of class A CpG ODN. Finally, considering the immune-compromised state of cancer patients, CpG ODN with enhanced immune activation property is needed by modifying nucleic acid sequences with immunostimulatory properties or incorporating immunogenic components in the delivery platforms. Ongoing clinical trials will elucidate more antitumor mechanisms of CpG ODN therapies by unmasking the roles of immune cells according to patient prognosis, further providing a precise rationale in designing novel CpG ODN candidates in the future.

## Figures and Tables

**Table 1:** CpG ODN monotherapy in current clinical trials.

Product	Type of CpG ODN	Indications	Route of Administration	Development Phase
CpG 7909	Class B	Renal Cell Carcinoma	Subcutaneously	Phase I/II (NCT00043407) [93]
		Cutaneous T-cell lymphoma	Subcutaneously	Phase I/II (NCT00043420) [94]
		Melanoma, Breast Neoplasm, Renal Cell Carcinoma, T-Cell Lymphoma, NSCLC	Subcutaneously and intravenously	Phase II (NCT00043368) [95]
		Melanoma	Subcutaneously	Phase II (NCT00070642) [96]
		Chronic Lymphocytic Leukemia	Subcutaneously and intravenously	Phase I (NCT00233506)
Litenimod	Class B	GBM	Intracerebral	Phase II (NCT00190424) [97,98]
IMO-2055	IMOs	Renal Cell Carcinoma	Subcutaneously	Phase II (NCT00729053)
GNKG168	Class B	B-CLL	Intravenously	Phase I (NCT01035216)
		ALL and AML	Intravenously	Phase I (NCT01743807)
MGN1703	dSLIM	Advanced Colorectal Carcinoma	Subcutaneously	Phase II (NCT01208194) [99]
		Advanced Colorectal Carcinoma	Subcutaneously	Phase III (NCT02077868) [99]
		Small Cell Lung Cancer	Subcutaneously	Phase II (NCT02200081) [100]
IMO-2125	IMOs	Solid Tumors	Intratumorally	Phase I (NCT03052205) [101]
		Melanoma	Intradermally	Phase II (NCT04126876)
AST-008	SNA	Solid Tumors	Intratumorally	Phase I/II (NCT03684785)

## Data Availability

The authors confirm that the data supporting the findings of this study are available within the article.

## References

[1] Urban-Wojciuk Z., Khan M.M., Oyler B.L., Fåhraeus R., Marek-Trzonkowska N., Nita-Lazar A., Hupp T.R., Goodlett D.R. (2019). The role of tlrs in anti-cancer immunity and tumor rejection. Front. Immunol..

[2] Kawai T., Akira S. (2007). Signaling to NF-κB by Toll-like receptors. Trends Mol. Med..

[3] Shinohara M.L., Lu L., Bu J., Werneck M.B.F., Kobayashi K.S., Glimcher L.H., Cantor H. (2006). Osteopontin expression is essential for interferon-α production by plasmacytoid dendritic cells. Nat. Immunol..

[4] Gotoh K., Tanaka Y., Nishikimi A., Inayoshi A., Enjoji M., Takayanagi R., Sasazuki T., Fukui Y. (2008). Differential requirement for DOCK2 in migration of plasmacytoid dendritic cells versus myeloid dendritic cells. Blood.

[5] Krieg A.M. (2008). Toll-like receptor 9 (TLR9) agonists in the treatment of cancer. Oncogene.

[6] Barberis M., Helikar T., Verbruggen P. (2018). Simulation of Stimulation: Cytokine Dosage and Cell Cycle Crosstalk Driving Timing-Dependent T Cell Differentiation. Front. Physiol..

[7] Krieg A.M. (2004). Antitumor applications of stimulating toll-like receptor 9 with CpG oligodeoxynucleotides. Curr. Oncol. Rep..

[8] Krieg A.M. (2006). Therapeutic potential of Toll-like receptor 9 activation. Nat. Rev. Drug Discov..

[9] Akdis M., Burgler S., Crameri R., Eiwegger T., Fujita H., Gomez E., Klunker S., Meyer N., O’Mahony L., Palomares O. (2011). Interleukins, from 1 to 37, and interferon-γ: Receptors, functions, and roles in diseases. J. Allergy Clin. Immunol..

[10] Paul S., Lal G. (2017). The Molecular Mechanism of Natural Killer Cells Function and Its Importance in Cancer Immunotherapy. Front. Immunol..

[11] Halle S., Halle O., Förster R. (2017). Mechanisms and Dynamics of T Cell-Mediated Cytotoxicity In Vivo. Trends Immunol..

[12] Kim H.-J., Cantor H. (2014). CD4 T-cell Subsets and Tumor Immunity: The Helpful and the Not-so-Helpful. Cancer Immunol. Res..

[13] Tiemessen M.M., Jagger A.L., Evans H.G., van Herwijnen M.J.C., John S., Taams L.S. (2007). CD4^+^CD25^+^Foxp3^+^ regulatory T cells induce alternative activation of human monocytes/macrophages. Proc. Natl. Acad. Sci. USA.

[14] Yang W.-C., Hwang Y.-S., Chen Y.-Y., Liu C.-L., Shen C.-N., Hong W.-H., Lo S.-M., Shen C.-R. (2017). Interleukin-4 Supports the Suppressive Immune Responses Elicited by Regulatory T Cells. Front. Immunol..

[15] Ye J., Livergood R.S., Peng G. (2013). The Role and Regulation of Human Th17 Cells in Tumor Immunity. Am. J. Pathol..

[16] Muranski P., Boni A., Antony P.A., Cassard L., Irvine K.R., Kaiser A., Paulos C.M., Palmer D.C., Touloukian C.E., Ptak K. (2008). Tumor-specific Th17-polarized cells eradicate large established melanoma. Blood.

[17] Hinrichs C.S., Kaiser A., Paulos C.M., Cassard L., Sanchez-Perez L., Heemskerk B., Wrzesinski C., Borman Z.A., Muranski P., Restifo N.P. (2009). Type 17 CD8^+^ T cells display enhanced antitumor immunity. Blood.

[18] Martin-Orozco N., Muranski P., Chung Y., Yang X.O., Yamazaki T., Lu S., Hwu P., Restifo N.P., Overwijk W.W., Dong C. (2009). T Helper 17 Cells Promote Cytotoxic T Cell Activation in Tumor Immunity. Immunity.

[19] Highfill S.L., Cui Y., Giles A.J., Smith J.P., Zhang H., Morse E., Kaplan R.N., Mackall C.L. (2014). Disruption of CXCR2-Mediated MDSC Tumor Trafficking Enhances Anti-PD1 Efficacy. Sci. Transl. Med..

[20] Peggs K.S., Quezada S.A., Chambers C.A., Korman A.J., Allison J.P. (2009). Blockade of CTLA-4 on both effector and regulatory T cell compartments contributes to the antitumor activity of anti–CTLA-4 antibodies. J. Exp. Med..

[21] Walker L.S.K., Sansom D.M. (2011). The emerging role of CTLA4 as a cell-extrinsic regulator of T cell responses. Nat. Rev. Immunol..

[22] Setoguchi R., Hori S., Takahashi T., Sakaguchi S. (2005). Homeostatic maintenance of natural Foxp3^+^ CD25^+^ CD4^+^ regulatory T cells by interleukin (IL)-2 and induction of autoimmune disease by IL-2 neutralization. J. Exp. Med..

[23] Cao X., Cai S.F., Fehniger T.A., Song J., Collins L.I., Piwnica-Worms D.R., Ley T.J. (2007). Granzyme B and Perforin Are Important for Regulatory T Cell-Mediated Suppression of Tumor Clearance. Immunity.

[24] Fleming V., Hu X., Weber R., Nagibin V., Groth C., Altevogt P., Utikal J., Umansky V. (2018). Targeting Myeloid-Derived Suppressor Cells to Bypass Tumor-Induced Immunosuppression. Front. Immunol..

[25] Li C., Liu T., Bazhin A.V., Yang Y. (2017). The Sabotaging Role of Myeloid Cells in Anti-Angiogenic Therapy: Coordination of Angiogenesis and Immune Suppression by Hypoxia. J. Cell. Physiol..

[26] Karakhanova S., Link J., Heinrich M., Shevchenko I., Yang Y., Hassenpflug M., Bunge H., von Ahn K., Brecht R., Mathes A. (2015). Characterization of myeloid leukocytes and soluble mediators in pancreatic cancer: Importance of myeloid-derived suppressor cells. Oncoimmunology.

[27] Wang Z., Jiang J., Li Z., Zhang J., Wang H., Qin Z. (2010). A Myeloid Cell Population Induced by Freund Adjuvant Suppresses T-cell−mediated Antitumor Immunity. J. Immunother..

[28] Hart K.M., Byrne K.T., Molloy M.J., Usherwood E.M., Berwin B. (2011). IL-10 Immunomodulation of Myeloid Cells Regulates a Murine Model of Ovarian Cancer. Front. Immunol..

[29] Azzaoui I., Uhel F., Rossille D., Pangault C., Dulong J., Le Priol J., Lamy T., Houot R., Le Gouill S., Cartron G. (2016). T-cell defect in diffuse large B-cell lymphomas involves expansion of myeloid-derived suppressor cells. Blood.

[30] Noman M.Z., Desantis G., Janji B., Hasmim M., Karray S., Dessen P., Bronte V., Chouaib S. (2014). PD-L1 is a novel direct target of HIF-1α, and its blockade under hypoxia enhanced MDSC-mediated T cell activation. J. Exp. Med..

[31] Kurreck J. (2003). Antisense technologies. Improvement through novel chemical modifications. Eur. J. Biochem..

[32] Malyala P., O’Hagan D.T., Singh M. (2009). Enhancing the therapeutic efficacy of CpG oligonucleotides using biodegradable microparticles. Adv. Drug Deliv. Rev..

[33] Wilson K.D., de Jong S.D., Tam Y.K. (2009). Lipid-based delivery of CpG oligonucleotides enhances immunotherapeutic efficacy. Adv. Drug Deliv. Rev..

[34] Rattanakiat S., Nishikawa M., Funabashi H., Luo D., Takakura Y. (2009). The assembly of a short linear natural cytosine-phosphate-guanine DNA into dendritic structures and its effect on immunostimulatory activity. Biomaterials.

[35] Bauer M., Heeg K., Wagner H. (1999). DNA activates human immune cells through a CpG sequence-dependent manner. Immunology.

[36] Krug A., Rothenfusser S., Hornung V., Jahrsdörfer B., Blackwell S., Ballas Z.K., Endres S., Krieg A.M., Hartmann G. (2001). Identification of CpG oligonucleotide sequences with high induction of IFN-α/β in plasmacytoid dendritic cells. Eur. J. Immunol..

[37] Verthelyi D., Ishii K.J., Gursel M., Takeshita F., Klinman D.M. (2001). Human Peripheral Blood Cells Differentially Recognize and Respond to Two Distinct CpG Motifs. J. Immunol..

[38] Ballas Z.K., Rasmussen W.L., Krieg A.M. (1996). Induction of NK activity in murine and human cells by CpG motifs in oligodeoxynucleotides and bacterial DNA. J. Immunol..

[39] Kawasaki T., Kawai T. (2014). Toll-Like Receptor Signaling Pathways. Front. Immunol..

[40] Rothenfusser S., Hornung V., Ayyoub M., Britsch S., Towarowski A., Krug A., Sarris A., Lubenow N., Speiser D., Endres S. (2004). CpG-A and CpG-B oligonucleotides differentially enhance human peptide–specific primary and memory CD8+ T-cell responses in vitro. Blood.

[41] Krepler C., Wacheck V., Strommer S., Hartmann G., Polterauer P., Wolff K., Pehamberger H., Jansen B. (2004). CpG Oligonucleotides Elicit Antitumor Responses in a Human Melanoma NOD/SCID Xenotransplantation Model. J. Investig. Dermatol..

[42] Ballas Z.K., Krieg A.M., Warren T., Rasmussen W., Davis H.L., Waldschmidt M., Weiner G.J. (2001). Divergent Therapeutic and Immunologic Effects of Oligodeoxynucleotides with Distinct CpG Motifs. J. Immunol..

[43] Reiniš M., Šímová J., Bubeník J. (2006). Inhibitory effects of unmethylated CpG oligodeoxynucleotides on MHC class I-deficient and -proficient HPV16-associated tumours. Int. J. Cancer.

[44] Ma Q., Zhou D., DeLyria E.S., Wen X., Lu W., Thapa P., Liu C., Li D., Bassett R.L., Overwijk W.W. (2017). Synthetic Poly(l-Glutamic Acid)-conjugated CpG Exhibits Antitumor Efficacy With Increased Retention in Tumor and Draining Lymph Nodes After Intratumoral Injection in a Mouse Model of Melanoma. J. Immunother..

[45] Qiu F., Maniar A., Quevedo Diaz M., Chapoval A.I., Medvedev A.E. (2011). Activation of cytokine-producing and antitumor activities of natural killer cells and macrophages by engagement of Toll-like and NOD-like receptors. Innate Immun..

[46] Huang L., Wang Z., Liu C., Xu C., Mbofung R.M., McKenzie J.A., Khong H., Hwu P., Peng W. (2017). CpG-based immunotherapy impairs antitumor activity of BRAF inhibitors in a B-cell-dependent manner. Oncogene.

[47] Ginzkey C., Eicker S.O., Marget M., Krause J., Brecht S., Westphal M., Hugo H.H., Mehdorn H.M., Steinmann J., Hamel W. (2010). Increase in tumor size following intratumoral injection of immunostimulatory CpG-containing oligonucleotides in a rat glioma model. Cancer Immunol. Immunother..

[48] Mutwiri G.K., Nichani A.K., Babiuk S., Babiuk L.A. (2004). Strategies for enhancing the immunostimulatory effects of CpG oligodeoxynucleotides. J. Control. Release.

[49] Zhao Q., Zhou R., Temsamani J., Zhang Z., Roskey A., Agrawal S. (1998). Cellular Distribution of Phosphorothioate Oligonucleotide Following Intravenous Administration in Mice. Antisense Nucleic Acid Drug Dev..

[50] Hartmann G., Weeratna R.D., Ballas Z.K., Payette P., Blackwell S., Suparto I., Rasmussen W.L., Waldschmidt M., Sajuthi D., Purcell R.H. (2000). Delineation of a CpG Phosphorothioate Oligodeoxynucleotide for Activating Primate Immune Responses In Vitro and In Vivo. J. Immunol..

[51] Gürsel M., Verthelyi D., Gürsel I., Ishii K.J., Klinman D.M. (2002). Differential and competitive activation of human immune cells by distinct classes of CpG oligodeoxynucleotide. J. Leukoc. Biol..

[52] Whitmore M., Li S., Falo L., Huang L. (2001). Systemic administration of LPD prepared with CpG oligonucleotides inhibits the growth of established pulmonary metastases by stimulating innate and acquired antitumor immune responses. Cancer Immunol. Immunother..

[53] Jiang J., Kong X., Xie Y., Zou H., Tang Q., Ma D., Zhao X., He X., Xia A., Liu P. (2019). Potent anti-tumor immunostimulatory biocompatible nanohydrogel made from DNA. Nanoscale Res. Lett..

[54] Heller L., Todorovic V., Cemazar M. (2013). Electrotransfer of single-stranded or double-stranded DNA induces complete regression of palpable B16.F10 mouse melanomas. Cancer Gene Ther..

[55] Hafner M., Zawatzky R., Hirtreiter C., Buurman W.A., Echtenacher B., Hehlgans T., Männel D.N. (2001). Antimetastatic Effect of CpG DNA Mediated by Type I IFN. Cancer Res..

[56] Chang L.-S., Leng C.-H., Yeh Y.-C., Wu C.-C., Chen H.-W., Huang H.-M., Liu S.-J. (2014). Toll-like receptor 9 agonist enhances anti-tumor immunity and inhibits tumor-associated immunosuppressive cells numbers in a mouse cervical cancer model following recombinant lipoprotein therapy. Mol. Cancer.

[57] Sun W., Fang M., Chen Y., Yang Z., Xiao Y., Wan M., Wang H., Yu Y., Wang L. (2016). Delivery System of CpG Oligodeoxynucleotides through Eliciting an Effective T cell Immune Response against Melanoma in Mice. J. Cancer.

[58] Browning R.L., Mo X., Muthusamy N., Byrd J.C. (2015). CpG oligodeoxynucleotide CpG-685 upregulates functional interleukin-21 receptor on chronic lymphocytic leukemia B cells through an NF-κB mediated pathway. Oncotarget.

[59] Bai L., Chen W., Li W., Zhou L., Niu C., Han W., Cui J. (2018). CpG Oligodeoxynucleotide-Induced Apoptosis of B-Lineage Acute Lymphoblastic Leukemia Is through C-MYC/P53/BAX Signaling Pathway. Blood.

[60] Liang X., Moseman E.A., Farrar M.A., Bachanova V., Weisdorf D.J., Blazar B.R., Chen W. (2010). Toll-like receptor 9 signaling by CpG-B oligodeoxynucleotides induces an apoptotic pathway in human chronic lymphocytic leukemia B cells. Blood.

[61] Wang S., Liu X., Qiao T., Zhang Q. (2017). Radiosensitization by CpG ODN7909 in an epidermoid laryngeal carcinoma Hep-2 cell line. J. Int. Med. Res..

[62] Yuan S., Qiao T., Li X., Zhuang X., Chen W., Chen X., Zhang Q. (2018). Toll-like receptor 9 activation by CpG oligodeoxynucleotideï¿½7909 enhances the radiosensitivity of A549 lung cancer cells via the p53 signaling pathway. Oncol. Lett..

[63] Li T., Hua C., Yue W., Wu J., Lv X., Wei Q., Zhu S., Zang G., Cui J., Liu Y.-J. (2020). Discrepant antitumor efficacies of three CpG oligodeoxynucleotide classes in monotherapy and co-therapy with PD-1 blockade. Pharmacol. Res..

[64] Liu X., Sun Y. (2019). Progress in radiotherapy and expression of PD-L1 in esophageal cancer. Hematol. Med. Oncol..

[65] Lemke-Miltner C.D., Blackwell S.E., Yin C., Krug A.E., Morris A.J., Krieg A.M., Weiner G.J. (2020). Antibody Opsonization of a TLR9 Agonist–Containing Virus-like Particle Enhances In Situ Immunization. J. Immunol..

[66] Vollmer J., Weeratna R., Payette P., Jurk M., Schetter C., Laucht M., Wader T., Tluk S., Liu M., Davis H.L. (2004). Characterization of three CpG oligodeoxynucleotide classes with distinct immunostimulatory activities. Eur. J. Immunol..

[67] Donhauser N., Helm M., Pritschet K., Schuster P., Ries M., Korn K., Vollmer J., Schmidt B. (2010). Differential Effects of P-Class Versus Other CpG Oligodeoxynucleotide Classes on the Impaired Innate Immunity of Plasmacytoid Dendritic Cells in HIV Type 1 Infection. AIDS Res. Hum. Retrovir..

[68] Cerkovnik P., Jezersek Novakovic B., Stegel V., Novakovic S. (2009). Class C CpG oligodeoxynucleotides as a single agent and in combination with radiotherapy efficiently delayed growth of subcutaneous B16F1 tumors. Innate Immun..

[69] Cerkovnik P., Novakovic B., Stegel V., Novakovic S. (2010). Tumor vaccine composed of C-class CpG oligodeoxynucleotides and irradiated tumor cells induces long-term antitumor immunity. BMC Immunol..

[70] Ghosh C.C., Heatherton K., O’Connell K., LaPorte J., Guha P., Cox B.F., Jaroch D., Katz S.C. Abstract 1793: Regional administration of Class C CpG Oligodeoxynucleotides results in superior intrahepatic TLR9 activation and immunomodulation compared to systemic infusion. AACR Poster.

[71] Li T., Wu J., Zhu S., Zang G., Li S., Lv X., Yue W., Qiao Y., Cui J., Shao Y. (2020). A Novel C Type CpG Oligodeoxynucleotide Exhibits Immunostimulatory Activity In Vitro and Enhances Antitumor Effect In Vivo. Front. Pharmacol..

[72] Wang S., Campos J., Gallotta M., Gong M., Crain C., Naik E., Coffman R.L., Guiducci C. (2016). Intratumoral injection of a CpG oligonucleotide reverts resistance to PD-1 blockade by expanding multifunctional CD8 + T cells. Proc. Natl. Acad. Sci. USA.

[73] Wittig B., Schmidt M., Scheithauer W., Schmoll H.-J. (2015). MGN1703, an immunomodulator and toll-like receptor 9 (TLR-9) agonist: From bench to bedside. Crit. Rev. Oncol. Hematol..

[74] Kapp K., Kleuss C., Schroff M., Wittig B. (2014). Genuine Immunomodulation With dSLIM. Mol. Ther. Nucleic Acids.

[75] Schmidt M., Anton K., Nordhaus C., Junghans C., Wittig B., Worm M. (2006). Cytokine and Ig-production by CG-containing sequences with phosphorodiester backbone and dumbbell-shape. Allergy.

[76] Schmidt M., Hagner N., Marco A., König-Merediz S.A., Schroff M., Wittig B. (2015). Design and Structural Requirements of the Potent and Safe TLR-9 Agonistic Immunomodulator MGN1703. Nucleic Acid Ther..

[77] Sheehan J.P., Lan H.-C. (1998). Phosphorothioate Oligonucleotides Inhibit the Intrinsic Tenase Complex. Blood.

[78] Henry S.P., Novotny W., Leeds J., Auletta C., Kornbrust D.J. (1997). Inhibition of Coagulation by a Phosphorothioate Oligonucleotide. Antisense Nucleic Acid Drug Dev..

[79] Shaw D.R., Rustagi P.K., Kandimalla E.R., Manning A.N., Jiang Z., Agrawal S. (1997). Effects of synthetic oligonucleotides on human complement and coagulation*. Biochem. Pharmacol..

[80] Agrawal S., Kandimalla E.R. (2007). Synthetic agonists of Toll-like receptors 7, 8 and 9. Biochem. Soc. Trans..

[81] Lee J., Chuang T.-H., Redecke V., She L., Pitha P.M., Carson D.A., Raz E., Cottam H.B. (2003). Molecular basis for the immunostimulatory activity of guanine nucleoside analogs: Activation of Toll-like receptor 7. Proc. Natl. Acad. Sci. USA.

[82] Yu D., Putta M.R., Bhagat L., Dai M., Wang D., Trombino A.F., Sullivan T., Kandimalla E.R., Agrawal S. (2008). Impact of Secondary Structure of Toll-Like Receptor 9 Agonists on Interferon Alpha Induction. Antimicrob. Agents Chemother..

[83] Wang D., Yu D., Agrawal S. (2015). Abstract A56: Intratumoral injection of IMO-2055, a novel Toll-like receptor 9 agonist, with ipilimumab induces a systemic tumor-specific immune response. Cancer Immunol. Res..

[84] Gonzales P., Chavira B., Swetel K., McCambridge K. (2007). The combination of IMO-2055, a synthetic agonist of toll-like receptor-9 (TLR9), and the multikinase inhibitor sorafenib tosylate demonstrate enhanced antitumor activity in a human non-small cell lung cancer xenograft model. Cancer Res..

[85] Wang D., Jiang W., Zhu F., Mao X., Agrawal S. (2018). Modulation of the tumor microenvironment by intratumoral administration of IMO-2125, a novel TLR9 agonist, for cancer immunotherapy. Int. J. Oncol..

[86] Bichat F., Maubant S., Mirjolet J.-F., Slos P., Krieg A.M., Morris A. (2017). Abstract 2669: Antitumor activity of the CMP-001 (TLR9 agonist) alone or combined with immune modulators in syngeneic tumor models. Proceedings of the Immunology.

[87] Cheng Y., Lemke-Miltner C.D., Wongpattaraworakul W., Wang Z., Chan C.H.F., Salem A.K., Weiner G.J., Simons A.L. (2020). In situ immunization of a TLR9 agonist virus-like particle enhances anti-PD1 therapy. J. Immunother. Cancer.

[88] Roberts T.C., Langer R., Wood M.J.A. (2020). Advances in oligonucleotide drug delivery. Nat. Rev. Drug Discov..

[89] Daniel W.L., Lorch U., Coates S., Bexon A.S., Mix S. (2019). Abstract CT044: AST-008, a TLR9 agonist spherical nucleic acid, activated NK cells, T cells, and cytokines in healthy subjects in a Phase I clinical trial. Cancer Res..

[90] Kandimalla E.R., Nallagatla S., Anderson B.R., Kang R. (2019). Abstract A136: AST-008, a novel TLR9 agonist SNA, induces abscopal antitumor effects in mouse tumor models. Cancer Immunol. Res..

[91] Griss J., Bauer W., Wagner C., Simon M., Chen M., Grabmeier-Pfistershammer K., Maurer-Granofszky M., Roka F., Penz T., Bock C. (2019). B cells sustain inflammation and predict response to immune checkpoint blockade in human melanoma. Nat. Commun..

[92] Kuo T.C., Harrabi O., Chen A., Sangalang E.R.B., Doyle L., Fontaine D., Li M., Han B., Pons J., Sim J. (2021). TAC-001, a Toll-Like Receptor (TLR9) Agonist Antibody Conjugate Targeting B cells, Promotes Anti-Tumor Immunity and Favorable Safety Profile Following Systemic Administration in Preclinical Models. AACR Present..

[93] Thompson J.A., Kuzel T., Drucker B.J., Urba W.J., Bukowski R.M. (2009). Safety and Efficacy of PF-3512676 for the Treatment of Stage IV Renal Cell Carcinoma: An Open-Label, Multicenter Phase I/II Study. Clin. Genitourin. Cancer.

[94] Kim Y.H., Girardi M., Duvic M., Kuzel T., Link B.K., Pinter-Brown L., Rook A.H. (2010). Phase I trial of a Toll-like receptor 9 agonist, PF-3512676 (CPG 7909), in patients with treatment-refractory, cutaneous T-cell lymphoma. J. Am. Acad. Dermatol..

[95] Link B.K., Ballas Z.K., Weisdorf D., Wooldridge J.E., Bossler A.D., Shannon M., Rasmussen W.L., Krieg A.M., Weiner G.J. (2006). Oligodeoxynucleotide CpG 7909 Delivered as Intravenous Infusion Demonstrates Immunologic Modulation in Patients With Previously Treated Non-Hodgkin Lymphoma. J. Immunother..

[96] Molenkamp B.G., Sluijter B.J.R., van Leeuwen P.A.M., Santegoets S.J.A.M., Meijer S., Wijnands P.G.J.T.B., Haanen J.B.A.G., van den Eertwegh A.J.M., Scheper R.J., de Gruijl T.D. (2008). Local Administration of PF-3512676 CpG-B Instigates Tumor-Specific CD8^+^ T-Cell Reactivity in Melanoma Patients. Clin. Cancer Res..

[97] Carpentier A., Laigle-Donadey F., Zohar S., Capelle L., Behin A., Tibi A., Martin-Duverneuil N., Sanson M., Lacomblez L., Taillibert S. (2006). Phase 1 trial of a CpG oligodeoxynucleotide for patients with recurrent glioblastoma1. Neuro-Oncol..

[98] Carpentier A., Metellus P., Ursu R., Zohar S., Lafitte F., Barrie M., Meng Y., Richard M., Parizot C., Laigle-Donadey F. (2010). Intracerebral administration of CpG oligonucleotide for patients with recurrent glioblastoma: A phase II study. Neuro-Oncol..

[99] Riera-Knorrenschild J., Arnold D., Kopp H.-G., Mayer F., Kroening H., Nitsche D., Kuhlmann J., Ziebermayr R., Andel J., Zurlo A. (2015). A subgroup with improved overall survival from the phase 2 IMPACT study: Maintenance therapy of metastatic colorectal cancer patients with the TLR-9 agonist MGN1703. J. Clin. Oncol..

[100] Thomas M., Ponce-Aix S., Navarro A., Riera-Knorrenschild J., Schmidt M., Wiegert E., Kapp K., Wittig B., Mauri C., Dómine Gómez M. (2018). Immunotherapeutic maintenance treatment with toll-like receptor 9 agonist lefitolimod in patients with extensive-stage small-cell lung cancer: Results from the exploratory, controlled, randomized, international phase II IMPULSE study. Ann. Oncol..

[101] Babiker H., Borazanci E., Subbiah V., Algazi A.P., Schachter J., Lotem M., Hendler D., Rahimian S., Minderman H., Haymaker C. (2020). 1031P Tilsotolimod engages the TLR9 pathway to promote antigen presentation and type I IFN signaling in solid tumours. Ann. Oncol..

[102] Zent C.S., Smith B.J., Ballas Z.K., Wooldridge J.E., Link B.K., Call T.G., Shanafelt T.D., Bowen D.A., Kay N.E., Witzig T.E. (2012). Phase I clinical trial of CpG oligonucleotide 7909 (PF-03512676) in patients with previously treated chronic lymphocytic leukemia. Leuk. Lymphoma.

[103] Banday A.H., Jeelani S., Hruby V.J. (2015). Cancer vaccine adjuvants—Recent clinical progress and future perspectives. Immunopharmacol. Immunotoxicol..

[104] Iribarren K., Bloy N., Buqué A., Cremer I., Eggermont A., Fridman W.H., Fucikova J., Galon J., Špíšek R., Zitvogel L. (2016). Trial Watch: Immunostimulation with Toll-like receptor agonists in cancer therapy. Oncoimmunology.

[105] Li J., Song W., Czerwinski D.K., Varghese B., Uematsu S., Akira S., Krieg A.M., Levy R. (2007). Lymphoma Immunotherapy with CpG Oligodeoxynucleotides Requires TLR9 Either in the Host or in the Tumor Itself. J. Immunol..

[106] Sagiv-Barfi I., Czerwinski D.K., Levy S., Alam I.S., Mayer A.T., Gambhir S.S., Levy R. (2018). Eradication of spontaneous malignancy by local immunotherapy. Sci. Transl. Med..

[107] Houot R., Levy R. (2009). T-cell modulation combined with intratumoral CpG cures lymphoma in a mouse model without the need for chemotherapy. Blood.

[108] Sharma P., Allison J.P. (2015). The future of immune checkpoint therapy. Science.

[109] Sarvaria A., Madrigal J.A., Saudemont A. (2017). B cell regulation in cancer and anti-tumor immunity. Cell. Mol. Immunol..

